# Tumor-associated immune cell infiltrate density in penile squamous cell carcinomas

**DOI:** 10.1007/s00428-022-03271-1

**Published:** 2022-01-13

**Authors:** Luca Hladek, Katrin Bankov, Jens von der Grün, Natalie Filmann, Melanie Demes, Stefan Vallo, Peter J. Wild, Ria Winkelmann

**Affiliations:** 1 Dr. Senckenberg Institute of Pathology, University Hospital Frankfurt, Goethe University, Frankfurt am Main, Germany; 2 Department of Radiotherapy and Oncology, University Hospital Frankfurt, Goethe University, Frankfurt am Main, Germany; 3 Institute of Biostatistics and Mathematical Modeling, Goethe University, Frankfurt am Main, Germany; 4 Institute of Medical Virology, Goethe University Frankfurt, Frankfurt am Main, Germany; 5 Urologie an der Zeil, Frankfurt am Main, Germany; 6 Frankfurt Institute for Advanced Studies (FIAS), Frankfurt am Main, Germany; 7 Wildlab, University Hospital Frankfurt MVZ GmbH, Frankfurt am Main, Germany

**Keywords:** Penile carcinomas, Tumor microenvironment, Tissue microarray, Antibodies

## Abstract

Penile squamous cell carcinomas are rare tumor entities throughout Europe. Early lymphonodal spread urges for aggressive therapeutic approaches in advanced tumor stages. Therefore, understanding tumor biology and its microenvironment and correlation with known survival data is of substantial interest in order to establish treatment strategies adapted to the individual patient. Fifty-five therapy naïve squamous cell carcinomas, age range between 41 and 85 years with known clinicopathological data, were investigated with the use of tissue microarrays (TMA) regarding the tumor-associated immune cell infiltrate density (ICID). Slides were stained with antibodies against CD3, CD8 and CD20. An image analysis software was applied for evaluation. Data were correlated with clinicopathological characteristics and overall survival. There was a significant increase of ICID in squamous cell carcinomas of the penis in relation to tumor adjacent physiological tissue. Higher CD3-positive ICID was significantly associated with lower tumor stage in our cohort. The ICID was not associated with overall survival. Our data sharpens the view on tumor-associated immune cell infiltrate in penile squamous cell carcinomas with an unbiased digital and automated cell count. Further investigations on the immune cell infiltrate and its prognostic and possible therapeutic impact are needed.

## Introduction

Penile neoplasias are orphan diseases in Europe which can be treated with good overall survival in case of early detection. Later stages demand for aggressive therapeutic approaches because of early lymphonodal spread [1]. Most of the penile neoplasias are squamous cell carcinomas (SCC) [2]. In the WHO classification of Tumours of the Urinary System and Male Genital Organs, there are three main categories for penile squamous carcinomas: Tumors not attributed to infections by human papillomavirus (HPV), tumors with association to HPV [3] and other rare carcinomas [2]. TNM stage, depth of invasion, lymphonodal status, perineural invasion and grading are the main prognostic factors known by now [4, 5]. Apart from these factors, a focus was drawn to the tumor-associated immune cell response in recent years. Attempts on measuring this answer have been made in different tumor entities to generate information on the patients’ outcome. Familiar examples are breast cancer [6], head and neck cancer [7], anal squamous cell carcinoma [8] and cervical cancer [9] to only name a few entities. For penile carcinomas, data is sparse. From a previous study, it is known that squamous cell carcinomas of the penis related to infection by HPV are associated with a different amount and composition of tumor infiltrating lymphocytes than non-HPV related squamous cell carcinomas of the penis [10]. Additionally, studies attempting to gain knowledge on the amount and prognostic impact of immune cells were conducted [11, 12]. In general, from an immunological point of view, tumors are separated into subgroups with low immune cell infiltrate, medium amount of immune cell infiltrate and high immune cell infiltrate [13].

With the advent of digital tools and artificial intelligence also into the field of pathology, an automated image analysis software for our investigations on squamous cell carcinomas of the penis was applied. The goal was to gain an unbiased view on the immune cell infiltrate of the penis in a cohort by not relying on individual pathologist’s assessments. This technique is reproducible and produces automated machine generated numbers. Correlation of these data to known tumor characteristics was aimed.

In our study, we investigated the tumor-associated immune cell infiltrate density (ICID) with three antibodies: CD3 and CD8 (T-cells) and CD20 (B-cells). For malignant melanoma, for example, presence of positive staining against these CD molecules is associated with differing patient outcome [14, 15]. Therefore, our aim was to measure the immune cells stained with the aforementioned antibodies and to generate data on its association with known clinicopathological features in a cohort of penile squamous cell carcinomas.

## Materials and methods

### Patients

Fifty-five therapy-naïve cases of invasive squamous cell carcinomas of the penis from the Dr. Senckenberg Institute of Pathology, Frankfurt am Main, Germany were investigated. All cases of penile squamous cell carcinomas that had sufficient tissue material and were diagnosed between October 2001 and October 2017 were selected from the biobank of the Dr. Senckenberg Institute of Pathology. From a total of 70 cases that were available, 15 were excluded in the process of case preparation and later analyses: Cases with insufficient tissue material (*n*=2) were excluded from the beginning. Further exclusions were made for cases in which no invasive carcinoma was present (*n*=6) and cases which were classified as metastases (*n*=2) and thus were not primary penile carcinomas. One case of penile basal cell carcinoma and one case of penile Paget’s disease, focally invasive (*n*=2), were also excluded. Cases in which no TMA cores were available, or all available cores were defined as missing, were also excluded (*n*=3). Tumor classification according to the current WHO classification system [16] was available for all patients, as well as infection status with human papillomavirus (HPV) gained via LCD array technique, as described before [17]. Clinicopathological characteristics are presented in Table 1.

**Table 1:** Clinicopathological characteristics of invasive penile carcinomas (*n*=55)

**Clinicopathological characteristics of invasive penile carcinomas**	**Categorization**	***n***	**%**
Age (range 41–85 years)	<65 years	26	47
	≥65 years	29	53
Tumor stage	pT1a	22	40
	pT1b	6	11
	pT2	22	40
	pT3	2	4
	pT4	2	4
	pTX	1	2
Nodal status	pN0	12	22
	pN>0	8	15
	pNX	35	64
Grading	G1	11	20
	G2	35	64
	G3	9	16
L	L0	43	78
	L1	12	22
V	V0	50	91
	V1	5	9
Pn	Pn0	49	89
	Pn1	6	11
R	R0	45	82
	R1	3	5
	R1(is)	3	5
	RX	4	7
Infiltration pattern	Infiltrative pattern	34	62
	Pushing margin	21	38
Morphological tumor type	Usual	49	89
	Basaloid	6	11
HPV status (LCD array)	Negative	22	40
	Low risk	2	4
	High risk	29	53
	Unknown	2	4
**Immunohistochemistry (IHC)**			
p16 IHC	Negative	26	47
	Positive	29	53

Tumor stage: Nomenclature according to current WHO classification system, Nodal stage—pN0: No tumor infiltrated lymph nodes detected in pathological examination, pN>0: Tumor infiltrated lymph nodes detected in pathological examination, pNX: Pathological lymphonodal status unknown, L: Lymphovascular invasion—L0: No lymphovascular invasion, L1: Lymphovascular invasion, V: Vascular invasion—V0: No vascular invasion, V1: Vascular invasion, Pn: Perineural invasion—Pn0: No perineural invasion, Pn1: Perineural invasion; R: Residual tumor—R0: No residual tumor, R1: Residual tumor; R1(is): Residual carcinoma in situ, RX: Unknown status. HPV: Human papillomavirus

Clinical follow-up was available for all patients. Overall survival was calculated from the date of biopsy to the date of last follow-up or death by any cause.

Formalin fixed paraffin embedded (FFPE) material resection specimens and biopsy probes were processed subsequently to diagnostic procedures. The specimens were surgical specimens. Material and pseudonymized patient data used in this study were provided by the University Cancer Center Frankfurt (UCT). Written informed consent was obtained from all patients, and the study was approved by the institutional review board of the UCT and the ethical committee at the University Hospital Frankfurt, according to the Declaration of Helsinki (project-numbers: SUG-02-2017, SUG-6-2018).

### Determination of disease representative areas

FFPE materials were routinely kept at room temperature (RT) and chilled on a cooling plate before sectioning. Current hematoxylin eosin (HE) stained sections underwent an additional pathology review to confirm and select disease representative regions of interest (ROI) including carcinoma, corresponding carcinoma in situ (CIS) and tumor adjacent physiological tissue.

### Slide digitization and production of a human tissue microarray (TMA)

HE stains were digitized using a 20× brightfield slide scanner (Pannoramic Scan II, 3D Histech, Budapest, Hungary). Annotations of defined ROI were set to a core diameter of 1 mm. The TMA was produced using the TMA Grand Master (3DHistech, Budapest, Hungary) and matching settings of 1 mm core diameter. Via slide overlay function, digital annotations were matched to the donor tissue and transferred to an empty recipient paraffin matrix. In total, three TMAs were produced, each containing well-characterized areas of the tumor and tumor adjacent physiological tissue as well as CIS to address tumor heterogeneity. Three ROIs for non-tumoral adjacent areas and six ROIs of carcinoma and corresponding CIS were defined per case. In the instances that the case provided insufficient tissue material for a total number of nine cores, less ROIs were defined, and therefore less than nine cores were produced.

### Immunohistochemistry

**Fig. 1:**
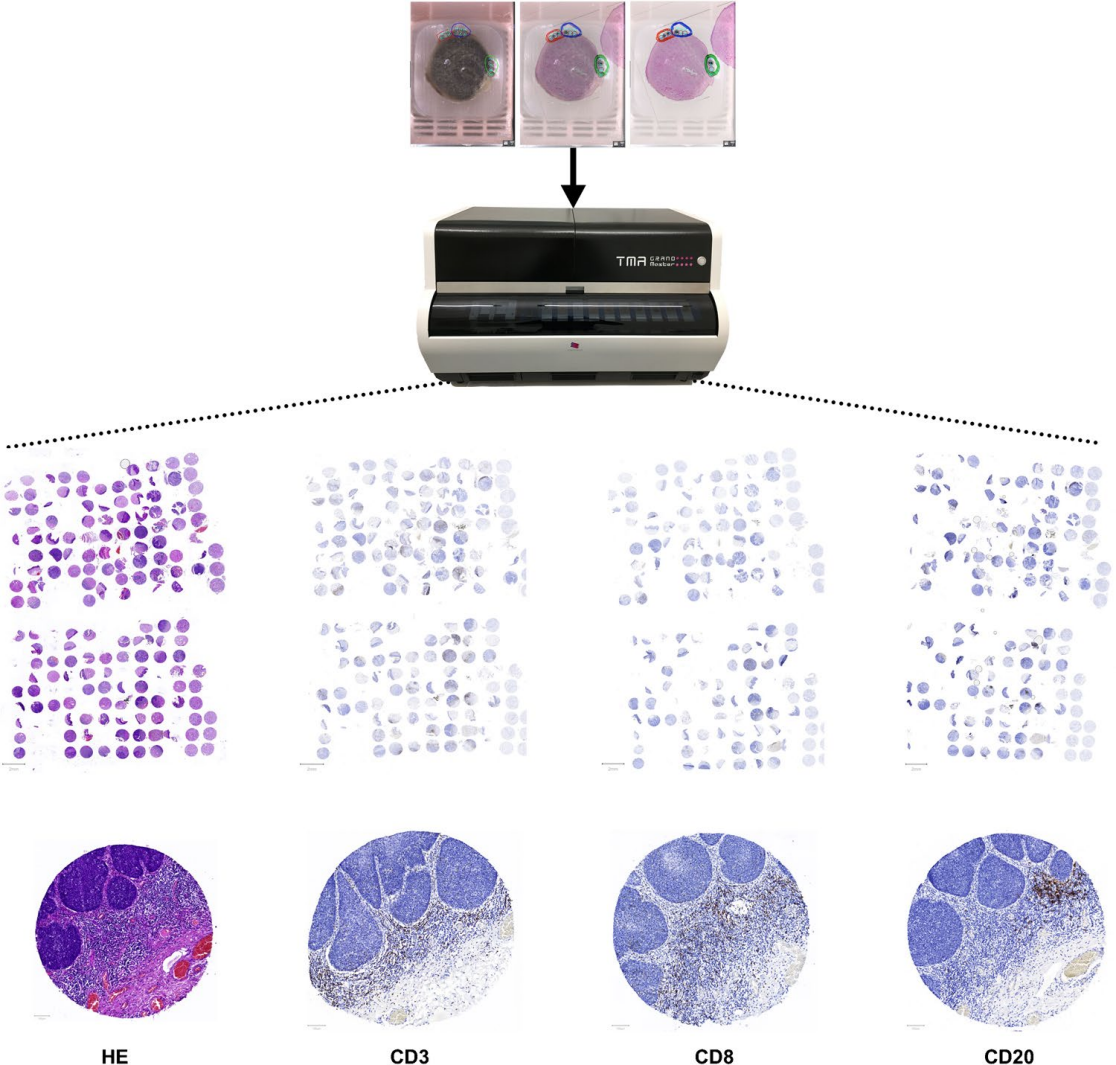
Schematic representation of the process of building the TMA and its digital evaluation. *The cores depicted on the bottom show the same core of the TMAs with different stains*

TMA sections were analyzed regarding the immune infiltrate of T-cells and B-cells by CD3, CD8 and CD20 targeting antibodies, respectively. Immunohistochemistry was performed using the DAKO FLEX-Envision Kit (Agilent, Santa Clara, CA, US) and the fully automated DAKO Omnis staining system (Agilent, Santa Clara, CA, US) according to manufacturer’s instruction. DAKO CD3 primary antibody (GA503, ready to use dilution, Agilent, Santa Clara, CA, US), CD8 primary antibody (GA623, ready to use dilution, Agilent, Santa Clara, CA, US) and CD20 primary antibody (GA604, ready to use dilution, Agilent, Santa Clara, CA, US) were used for immunohistochemical epitope staining applied for 30 min after heat-induced epitope retrieval in pH6 at 97 °C. Epitope visualization was done by DAKO EnVision™ FLEX DAB+ Substrate Chromogen System (Agilent, Santa Clara, CA, US) resulting in brown-ish cytoplasmic, membranous signal. Nuclear counterstain was done using DAKO hematoxylin solution (Agilent, Santa Clara, CA, US). Slide staining was supported by Nina Becker (technical assistant at Dr. Senckenberg Institute of Pathology, University Hospital Frankfurt, Frankfurt am Main and University Cancer Center, Goethe University Frankfurt am Main). The stained TMA slides were digitized as mentioned in the previous paragraph (Pannoramic Scan II, 3D Histech, Budapest, Hungary). The TMA process and its digital evaluation are shown in a schematic representation in Fig. 1.

### Evaluation of the stainings

Digitized TMA slides were analyzed using QuPath (Version 0.2.0), an open source software for image analysis [18]. Preprocessing and tissue and cell detection were conducted as suggested in the software’s documentation. Optical quality control and pathology review were performed. Missing TMA cores were defined as such, if not yet automatically identified by QuPath, and artifacts were removed. Per TMA core, QuPath returned absolute numbers of positively stained cells as well as a density value that counted this number of positive cells in relation to the detected total tissue area of the TMA core, resulting in a value that indicated the tumor-associated immune cell infiltrate density (ICID) for the specific stain for each core as the number of positive cells per mm^2^.

Per stain, a mean density value for every patient for each tumor and tumor adjacent physiological tissue was calculated separately. TMA cores containing tissue material from CIS regions were assigned to the tumor group. Six mean density values per patient were generated for tumor and tumor adjacent physiological tissue per stain. For correlations and survival analysis, only the densities in neoplastic tissues were used. Data for intra- and peritumoral immune cells were not generated separately, as the purpose was to assess the distinction between tumor and tumor adjacent physiological tissue.

The computed density means for neoplastic tissues were used to calculate a median split that divided the samples into two groups. For the three stains separately, mean density values above the median mean density value were grouped in the high group, mean densities smaller than the median grouped in the low category.

Furthermore, ratios of the aforementioned calculated density means were generated by dividing them by themselves. The calculated density ratios were also grouped into groups with high or low ratios, according to their relation to the median as outlined before.

### Statistical analyses

Statistical analyses were conducted with R Version 4.1.0 [19] in R Studio, Version 1.4.1717 [20]. Used packages include inter alia the ggplot2 v3.3.5 [21], psych v2.1.6 [22] and survival v3.2-11 [23] packages. The full reproducible code used for the analyses can be found in the Online Resource 1.

**Fig. 2:**
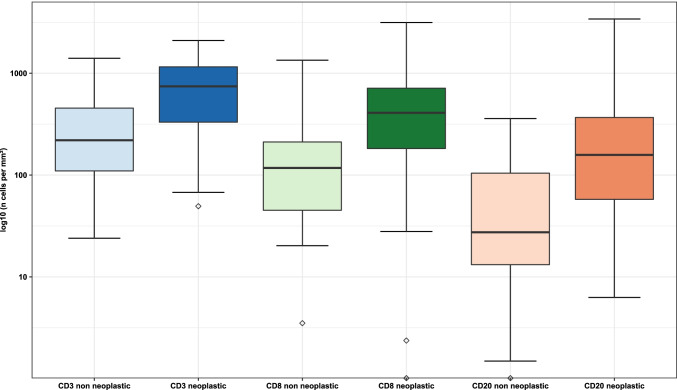
Graphical representation of differences in tumor-associated immune cell infiltrate density (ICID) between normal and neoplastic tissue. *Density of CD3-positive cells differs significantly between normal and neoplastic tissue (p = 0.0007); the same is true for CD8-positive cells (p = 0.0387) and CD20-positive cells (p = 0.0025). Furthermore, there are significant differences in the densities of CD3, CD8 and CD20-positive cells in neoplastic tissue: The difference between CD3-positive ICID and CD8-positive ICID is significant with p = 0.0001, the difference between CD3-positive ICID and CD20-positive ICID with p < 0.0001 and the difference between CD8-positive ICID and CD20-positive ICID with p = 0.0061*

Differences between the groups shown in Fig. 2 were calculated using Kruskal-Wallis and paired exact Wilcoxon-Pratt Signed-Rank Test with the reported *p* values corrected by Bonferroni’s method. Correlations between ICID and clinicopathological characteristics were calculated using either Spearman’s rank correlation for correlating interval type data with other interval type data, point-biserial correlation for correlating interval type data with dichotomous variables or using Kendall’s correlation as equivalent to the Jonckheere-Terpstra test for correlating interval type data with ordinal type data. No Bonferroni correction was applied to the correlation *p* values due to the exploratory nature of the correlation calculations.

Overall survival was analyzed by Kaplan-Meier estimators. Patients were censored at the point of their last clinical follow-up visit. Statistical significance was considered for *p*<0.05.

## Results

### Tumor and non-tumor tissue show different ICID

**Fig. 3:**
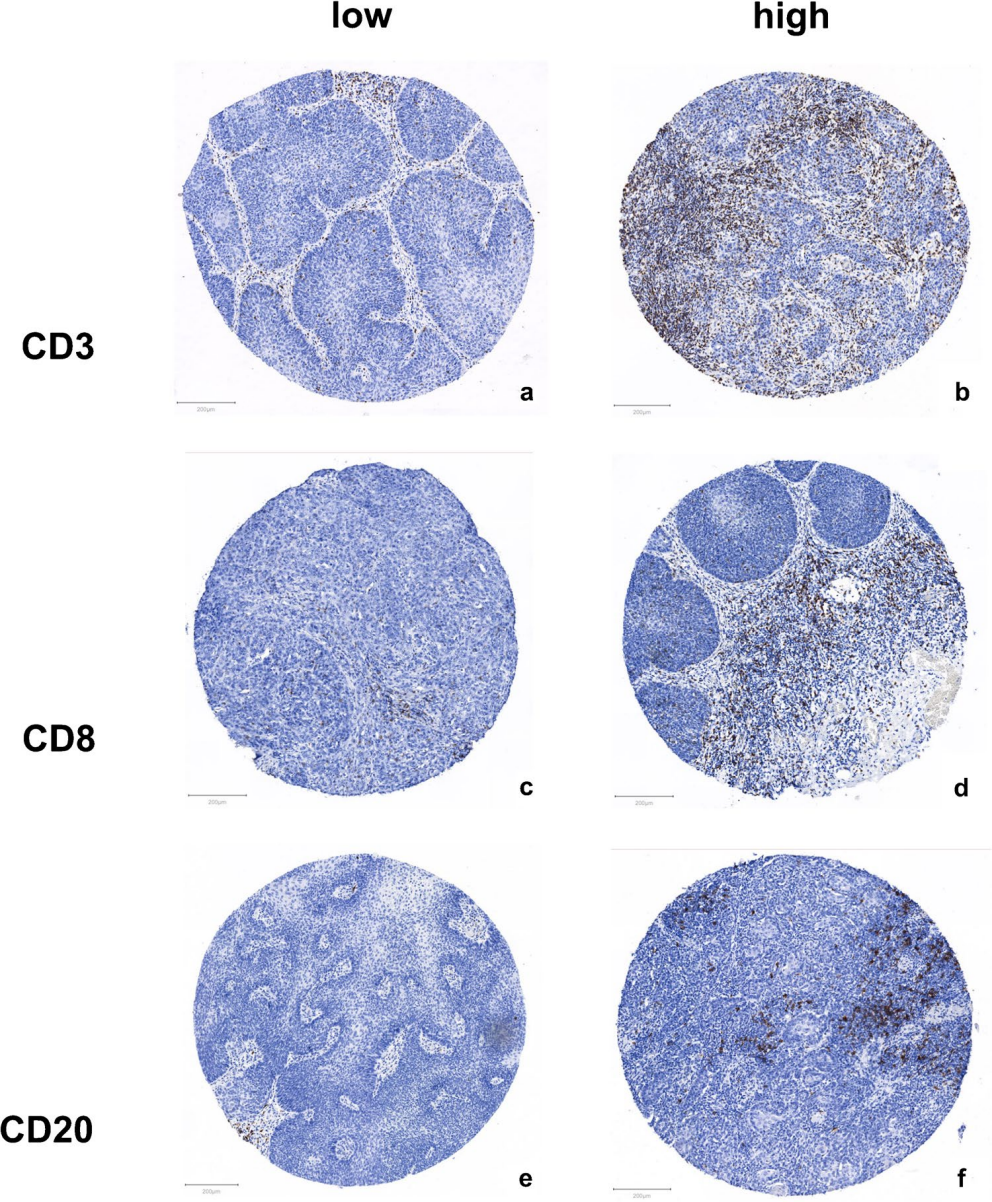
Visual impression of the different immune cell infiltrate in exemplary TMA cores with low vs high immune cell infiltrate density. TMA cores were stained using immunohistochemistry antibodies as described in the text. ***a*** *shows a core with low CD3-positive cell count (317.08 pos. cells per mm*^*2*^*),* ***b*** *shows a core with high CD3-positive cell count (3339.45 pos. cells per mm*^*2*^*). Median CD3 cell density for all patients was 512.24 pos. cells per mm*^*2*^*.* ***c*** *shows a core with low CD8-positive cell count (188.25 pos. cells per mm*^*2*^*),* ***d*** *shows a core with high CD8-positive cell count (1313.28 pos. cells per mm*^*2*^*). Median CD8 cell density for all patients was 217.53 pos. cells per mm*^*2*^*.* ***e*** *shows a core with low CD20-positive cell count (34.10 pos. cells per mm*^*2*^*),* ***f*** *shows a core with high CD20-positive cell count (768.07 pos. cells per mm*^*2*^*). Median CD20 cell density for all patients was 80.78 pos. cells per mm*^*2*^*. Density values are not to be interpreted as absolute numbers but to be looked at in relation to one another as they are highly specific to methods and sample material. Consequently, absolute numbers displayed here might not be directly comparable to other data*

Comparing tumor and non-tumor tissue of our cohort of invasive squamous cell carcinomas of the penis, statistically significant differences in the distribution of the ICID stained via CD3, CD8 and CD20 antibodies were detected (Fig. 3). For all three stains, non-neoplastic tissue showed lower amounts of the assessed types of immune cells than corresponding neoplastic tissue (Fig. 2). Additionally, we found that the densities of the three stained positive immune cells differed significantly in neoplastic tissue (Fig. 2).

### Correlation of ICID with clinicopathological characteristics

Recorded tumor and clinicopathological characteristics were correlated with the attained ICID values separately as described above. Data are presented in Table 2. A significant correlation was found between lower tumor stage and higher density of CD3-positive ICID (*p* = 0.03, *r* = −0.31). No significant correlations with the ICID were found for age group, lymphonodal status, tumor grading, lymphatic vessel invasion status, vascular invasion status, perineural infiltration (Pn), resection boundaries status, infiltration pattern, HPV status and p16 status. Basaloid tumor type related to a higher density of CD8-positive cells (*p* = 0.02, *r* = 0.33) in this cohort. There were no significant correlations between the calculated density ratios and recorded tumor characteristics, as shown in Supplementary Table 1.

**Table 2:** Correlations of immune cell infiltrate density in neoplastic tissue with clinicopathological characteristics in invasive penile carcinomas (*n* = 55)

**Clinicopathological characteristics of invasive penile carcinomas**	**Categorization**	**Correlation with CD3 density in neoplastic tissue**	***p*** **value**	**Correlation with CD8 density in neoplastic tissue**	***p*** **value**	**Correlation with CD20 density in neoplastic tissue**	***p*** **value**
Age (range 41–85 years)	< 65 years	0.09	NS	0.15	NS	0.03	NS
	≥ 65 years						
Tumor stage	pT1a	**−0.31**	0.03*	−0.18	NS	-0.10	NS
	pT1b						
	pT2						
	pT3						
	pT4						
	pTX						
Nodal status	pN0	−0.30	NS	−0.04	NS	−0.35	NS
	pN > 0						
	pNX						
Grading	G1	0.05^a^	NS	0.19^a^	NS	0.00^a^	NS
	G2						
	G3						
L	L0	−0.02	NS	−0.02	NS	−0.10	NS
	L1						
V	V0	−0.02	NS	0.10	NS	0.02	NS
	V1						
Pn	Pn0	0.03	NS	−0.01	NS	−0.12	NS
	Pn1						
R	R0	−0.06	NS	−0.16	NS	0.20	NS
	R > 0						
	RX						
Infiltration pattern	Infiltrative pattern	−0.13	NS	−0.10	NS	−0.13	NS
	Pushing margin						
Morphological tumor type	Usual	0.15	NS	**0.33**	0.02*	0.13	NS
	Basaloid						
HPV status (LCD array)	Negative or low risk	0.01	NS	0.11	NS	0.24	NS
	High risk						
	Unknown						
**Immunohistochemistry (IHC)**							
p16 IHC	Negative	0.03	NS	0.13	NS	0.25	NS
	Positive						

Tumor stage: Nomenclature according to current WHO classification system, Nodal stage—pN0: No tumor infiltrated lymph nodes detected in pathological examination, pN>0: Tumor infiltrated lymph nodes detected in pathological examination, pNX: Pathological lymphonodal status unknown, L: Lymphovascular invasion—L0: No lymphovascular invasion, L1: Lymphovascular invasion, V: Vascular invasion—V0: No vascular invasion, V1: Vascular invasion, Pn: Perineural invasion—Pn0: No perineural invasion, Pn1: Perineural invasion; R: Residual tumor—R0: No residual tumor, R > 0: Residual invasive tumor or residual carcinoma in situ, RX: Unknown status. HPV: Human papillomavirus

NS non-significant *p* value

*Significant *p* value

^a^Correlation calculated using Kendall’s correlation as an equivalent to Jonckheere-Terpstra test

### Correlation of ICID and outcome

**Fig. 4:**
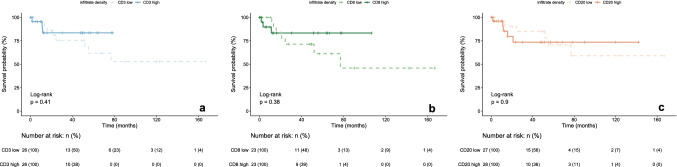
Kaplan-Meier plots showing the results of the survival analysis for groups of high vs low immune cell infiltrate density in neoplastic tissue for CD3-positive cells (**a**), CD8-positive cells (**b**) and CD20-positive cells (**c**). *There was no statistically significant difference in survival between groups of high vs low CD3, CD8 and CD20-positive ICID in our cohort of penile neoplasias*

There was no significant association between overall survival (OS) and ICID groups (low versus high ICID) measured by CD3, CD8 and CD20-positive cells (Fig. 4). Similarly, a significant association of the ICID ratios and their groups with OS was not shown (Supplementary Figure 1). A trend towards differing survival can be estimated. Additionally, correlations for the density ratios were calculated. Results are shown in the Supplementary Table 1. There was no significant association of the ICID ratios in relation to clinicopathological characteristics in the cohort.

## Discussion

The tumor microenvironment of penile squamous cell carcinomas is complex. Attempts to characterize the immune cells have been undertaken in different retrospective cohorts [11, 12, 24].

It is known that the penis is a location of immune response as it is the entry for sexually transmitted diseases [25]. In the setting of carcinomas, an immunologic role of the cancer cells had been postulated. Therefore, studies were undertaken investigating the cancer ‘immune profile’ based on antibodies to characterize subsets of immune cells that accumulate peri- and intratumoral [26]. Especially for CD3 and CD8, studies described differences in the risk of death or progression in some tumors [26]. It is also known that, additionally to T-cells, memory B-cells, as a driver of adaptive immunity for humoral response, are residing in the penis [25]. The interaction between B- and T-cells is a substantial part of immunity. Therefore, in recent years, studies were initiated to investigate B-cells as part of the tumor microenvironment [27, 28]. That is why we chose to investigate the tumor-associated immune cell infiltrate by means of the antibodies against CD3, CD8 and CD20.

The ICID of squamous cell carcinomas of the penis was investigated via image analysis software based on TMAs. An advantage of this study is the computer-based image analysis software, creating an unbiased view on the tumors. Moreover, TMA replicates were included representing neoplastic, CIS and non-neoplastic ROI of different tumor regions allowing for tumor heterogeneity assessment. By examining the density of the different immune cells rather than their absolute numbers, comprehensive and comparable data for characterizing the (immune) tumor microenvironment in penile carcinomas were retrieved.

There was a statistically significant association between neoplastic tissue and increased ICID stained with antibodies against CD3, CD8 and CD20. It is well known that tumors induce an immunologic response with consecutive interaction by the host [26]. Especially in HPV-associated cancers, such as penile squamous cell carcinomas, the amount of tumor-associated immune cells is increased [10]. Studies indicate a survival benefit for patients with increased amount of immune cells in association with the tumor [26]. This fact is used for anti-cancer immune therapies targeting specific immune cells. A popular example is the PD-L1 antibody which generates positive effects on patients’ OS in a growing amount of tumor entities. It is known that PD-L1 is expressed in penile carcinomas [17].

In this study, the ICID of invasive squamous cell carcinomas of the penis was investigated with antibodies against CD3, CD8 and CD20.

CD20 is a marker that can be proven on B-cells prior to their differentiation to plasma cells. It is expressed on naïve and memory B-cells. Most of the studies investigating B-cells in human cancer found a positive prognostic effect for CD20-positive B-cells in cancer types [29]. In one study investigating penile squamous cell carcinomas, the effect was described as neutral [11]. There might be a bias caused by the small number of penile carcinomas investigated compared to the large number of patients in the breast cancer cohort. Indirectly the effect of B-cells is already under investigation in penile squamous cell carcinomas through blocking of inhibitory signals in the PD-1/PD-L1 pathway in studies [24]. This pathway is important for interactions between plasma cells and T follicular helper cells [30]. Expecting CD20-positive B-cells to differentiate towards plasma cells, future studies may utilize double staining for a CD20 and a plasma cell marker to elucidate the amount and prognostic impact of plasma cells in invasive penile squamous cell carcinomas of the penis to further provide evidence for anti-PD-L1 therapies.

CD3 is expressed on T-cells. Some CD3-positive T-cells also express CD8. Non-anergic CD8-positive cells are cytotoxic T-cells with the capability of killing tumor cells [26]. In a meta-analysis, CD3 and CD8-positive cells had a positive effect on prognosis in different studies, also dependent on the study size [26]. In this study, a significant effect on overall survival regarding CD3-positive cells was not shown but might be readable from the risk tables, which is difficult to determine with the sample size of this cohort. This would be in line with a recent study by our group, which showed that the amount of immune cells, semi-quantitatively estimated, is associated with tumor stage and therefore indirectly with survival [17].

In recent years, attention was drawn to ratios between subsets of immune cells as predictive values. This approach enhances the view towards the immune system as a team player. There are conflicting data towards the predictive value on the ratio of CD3 and CD8-positive cells in different cancer types [26]. Therefore, further studies are needed applying more antibodies to understand the complex interactions and dynamics of the immune response and their possible targets for specific therapies.

Other studies reported different effects of survival depending on the localization of the immune cells (intratumoral and stromal) in specific tumor entities [31]. Possibly, unraveling those compartments would reveal statistically significant *p* values in our cohort. Nevertheless, it is not feasible in routine use and was therefore neither attempted nor intended.

In our cohort, the density of CD8-positive T-cells was associated with basaloid tumor cell morphology. That is not surprising since basaloid morphology is related to HPV infection and therefore to an increased amount of immune cells [10]. However, a significant correlation between HPV status and density of CD8-positive cells was not shown, which is most likely due to the sample size. A significant correlation should be present in a larger cohort, which further illustrates the need for additional studies.

Other parameters such as age group or lymphonodal status showed no association with the measured ICID. Interestingly, we were not able to show an association between overall survival and CD8-positive T-cells in our cohort, as did others [32]. This might also relate to sample size.

Sample size was a limiting factor for obtaining statistically significant correlations in this study, which is a frequent challenge in studies on penile cancer due to the small number of cases throughout Europe. Stronger correlations should be expected in studies with even larger sample sizes, especially for survival analyses and correlations of immune cell infiltrate densities with clinical data such as grading and staging categories.

Nevertheless, this cohort, with regard to the selection of methods and the resulting low bias and consideration for tumor heterogeneity, still allows a comprehensive review of the immune tumor microenvironment in penile cancer.

Future studies are needed to further describe and understand the tumor-associated immune cell infiltrate in penile squamous cell carcinomas and to find new targets for antitumor therapy regimens in this mutilating disease.

## Conclusion

Neoplastic tissue in penile squamous cell carcinomas is associated with an increased density of immune cells as shown with antibodies against CD3, CD8 and CD20. Therefore, it was shown, that not only T-cells are increased in number and are possible targets to potential anti-tumor therapies, but also CD20-positive B-cells. This enhances the need to further elucidate the role of B-cells in penile squamous cell carcinomas to eventually generate possible new target strategies for this neoplasm.

Further studies are needed to generate insight on the activation status of the immune cells. Maybe in that way new strategies can be elucidated facilitating this microenvironment in order to generate an increased anti-tumor effect of the immune cell infiltrate.

## Abbreviations

ICID: Tumor-associated immune cell infiltrate density
SCC: Squamous cell carcinoma(s)
FFPE: Formalin fixed paraffin embedded
HE: Hematoxylin and eosin
UCT: University Cancer Center Frankfurt
ROI: Region(s) of interest
TMA: Tissue microarray
CIS: Carcinoma in situ
HPV: Human papillomavirus
OS: Overall survival
